# The influence of body mass index (BMI) on the reproducibility of surface topography measurements

**DOI:** 10.1186/1748-7161-7-S1-O18

**Published:** 2012-01-27

**Authors:** P Knott, S Mardjetko, D Tager, R Hund, S Thompson

**Affiliations:** 1 Rosalind Franklin University North Chicago, USA; 2 Illinois Bone and Joint Institute, USA; 3 Northwestern University, USA

## Background

Surface Topography can be used to evaluate patients with spinal deformity, especially adolescents with scoliosis in whom a reduced number of radiographic evaluations is desired. The Formetric 4D (Diers International GmbH, Schlangenbad, Germany) is a surface topography system that is able to identify anatomical landmarks and construct a 3-dimentional model of the spine using only surface features. One would guess that a slender patient with easily identifiable bony landmarks would be an ideal patient for this system, and that a patient with a higher Body Mass Index (BMI) would be more difficult to measure [1-5].

## Materials and methods

In this study, fourteen female patients were measured 30 times each to evaluate the reproducibility of the Formetric measurements. The patients ranged in BMI from 16.9 to 29.0, and the reproducibility of each of the Formetric parameters was correlated to BMI.

## Results

Results showed that there was not a strong correlation between any of the individual surface topography parameters and the BMI. The reproducibility of the calculated scoliosis curve did correlate with BMI, however, (r = 0.65) and this correlation was significant (p = 0.012), showing that the higher the patient's BMI, the more variability was present in scoliosis angle calculations.

## Conclusions

Overall, the reproducibility of the Formetric 4D was very good even in patients with higher BMI. The patient with the highest BMI (29) still had Formetric measurements that were +/- only 4.6 degrees for scoliosis curve calculations.
